# Oral health and depressive symptoms among older adults in urban China: a moderated mediation model analysis

**DOI:** 10.1186/s12877-022-03542-1

**Published:** 2022-10-28

**Authors:** Qian Sun, Youwei Wang, Qingsong Chang

**Affiliations:** 1 Department of Social Security, School of Public Administration, Hebei University of Economics and Business, Shijiazhuang, China; 2 Hebei Collaborative Innovation Center on Urban-rural Integrated Development, Shijiazhuang, China; 3 Sau Po Centre on Ageing, The University of Hong Kong, Hong Kong, Hong Kong; 4 School of Sociology & Anthropology, Sun Yat-sen University, Guangzhou, Guangdong Province China; 5 School of Sociology and Anthropology, Xiamen University, Xiamen, Fujian Province China

**Keywords:** Ageing process, Daily dietary satisfaction, Body mass index, Health ageing

## Abstract

**Background:**

This study explored the association between oral health and depression occurs via daily dietary satisfaction as a mediator, and that body mass index could moderate the path between daily dietary satisfaction and depression.

**Methods:**

Data for this research were derived from a community survey adopting quota sampling in the cities of Tianjin and Shijiazhuang in mainland China in 2020 (*N* = 781). The moderated mediation model was tested by using bootstrapping with resampling strategies, and the Johnson-Neyman technique was used to visualize the moderating effect of body mass index.

**Results:**

A significant negative association between oral health and depression has been indicated (B = −0.22, SE = 0.11, 95%CI [− 0.44, − 0.01]), and dietary satisfaction partially mediated the relationship between oral health and depression (B = −0.04, SE = 0.02, 95%CI [− 0.09, − 0.002]). The path was moderated by body mass index, and the effect of dietary satisfaction on depression was much greater in people with relatively low body mass index.

**Conclusions:**

This study present evidence for policymakers and researchers that strategies to enhance oral health and daily dietary satisfaction could be important for preventing depression in Chinese older adults, and especially for the relatively fitter older groups with lower body mass index.

**Supplementary Information:**

The online version contains supplementary material available at 10.1186/s12877-022-03542-1.

## Background

Depression is a pervasive mental disorder worldwide, with an estimated 3.8% of the population affected, including 5.7% of adults older than 60 years [1]. Since 2013, depression has been identified as the fourth leading cause of disability in older adults [2]. In China, depression has also become a major public health issue affecting older residents. According to the latest data from the China Health and Retirement Longitudinal Study (CHARLS), approximately 36.9% of older adults reported depressive symptoms (CHARLS Research Team, 2018). The occurrence of depression in late life was significantly associated with an elevated caregiving burden, an elevated possibility of suicide, and an increased use of healthcare [3]. Predictors of depression among Chinese older adults include, but are not limited to, demographic, physical, economic, cognitive, and social factors [4–6]. However, as an important aspect of general health status, the relationship between oral health and depressive symptoms among older adults has rarely been studied in Chinese communities.

Nevertheless, studies from non-Chinese communities have thoroughly documented oral health as a significant factor leading to depression. For example, toothache, periodontal bleeding, tooth loss, and edentulism have been indicated as risk factors of depression [7, 8]. A possible explanation of these findings is that oral health is an important contributor to the life circumstances of older adults. Scholars have found that oral diseases can predict older people’s lifestyle and subjective well-being as a result of problems with food selection, nutritional status, facial profile, and social interaction causing depressive symptoms [7, 9].

Among the aforementioned challenges, older adults with poor oral health are particularly vulnerable to changes in food preferences and deficient nutritional intake in their daily diet [10, 11]. Older people who have difficulties on chewing or biting comfortably are less likely feel satisfied with their daily dietary intake. In Chinese society, according to the viewpoint of Chinese Medicine, it is believed that a poor diet causes diseases. An old Chinese proverb says ‘a good appetite is a blessing for older adults.’ Therefore, we argue that daily dietary satisfaction provides a mediation mechanism that helps explain the correlation between oral health and depressive symptoms among Chinese older adults. However, the mediating effect of daily dietary satisfaction on the relationship between oral health and depressive symptoms is not well understood in the current literature.

In addition, available studies have revealed that depression and other relevant indicators (such as quality of life, subjective well-being, and life satisfaction) fluctuated with body mass index [12–15]. Overweight older individuals were significantly more likely to be vulnerable to depression [16] than their counterparts with a lower body mass index. This may reflect a biological mechanism that weight gain and obesity involved increased risks of inflammation, the hypothalamic-pituitary-adrenal axis dysregulation, diabetes mellitus, and insulin resistance, which could correlate with depressive symptoms closely [14]. Apart from biological mechanism, social-psychological mechanism underlying the moderating role of body mass index should be outlined. It is necessary to point out that individuals with different BMIs display diverse food structures and dietary preferences, which may have influence on the path between dietary satisfaction and depressive symptoms. There was research showing that a dietary intake pattern characterized by higher consumption levels of cereals, nuts, vegetables and fruits (which required healthier oral health conditions) was more prevalent among older adults with lower body mass index than among those in the obese group [17]. Taken both biological and social-psychological mechanisms together, a more comprehensive analytic framework should be considered when exploring the mediating effect of dietary satisfaction on the relationship between oral health and depressive symptoms, and we assume that the relationship between daily dietary satisfaction and depressive symptoms differs across various levels of body mass index.

Overall, although poor oral health has been shown to be positively associated with depressive symptoms, research conducted in the Chinese context has been very limited. Whereas China has shown a similar aging process to the global trend of aging, Chinese older individuals may have different patterns in terms of health and of risk factors for well-being due to their unique lifestyle and culture. Therefore, it is worthwhile to investigate the association between oral health and depressive symptoms among Chinese older adults. Moreover, we also speculate that an individual’s daily dietary satisfaction is an important mediator, in conjunction with the moderating effect of body mass index. Thus, when we examine oral health as a risk factor for depressive symptoms, we will include the heretofore rarely investigated moderated mediation effect of daily dietary satisfaction. Consequently, the aims of this study were to understand how oral health relates to depressive symptoms via a moderated mediation model. We postulated that the mediator (daily dietary satisfaction) and the moderator (body mass index) would represent a comprehensive interrelationship and would provide convincing new mechanisms for the association between oral health and depressive symptoms among older Chinese adults.

To be more specific, we proposed the hypotheses as follows:

- *Hypothesis 1*. There is an association between oral health and depressive symptoms in Chinese older adults (total effect c’).
- *Hypothesis 2*. Oral health has a specific, indirect effect on depressive symptoms through dietary satisfaction (mediation effect a-b).
- *Hypothesis 3*. Body mass index has a specific moderating effect on the path between dietary satisfaction and depressive symptoms (moderation effect b-d).

## Methods

### Data and sampling

The data for this study were derived from a community survey of China, in 2020. A quota sampling method was used to select a sample from Tianjin City and Shijiazhuang City in Hebei Province, China. First, five districts were randomly selected in each city. Second, two communities were randomly selected in each district, for a total of 10 communities in each city. Forty respondents aged 60 years and older were selected in each community. Age and gender ratio of the respondents were controlled in accord with their statistical representation in the latest local demographic data. The respondents had to meet the following criteria: (1) have local household registration status; (2) be aged 60 years or older; (3) have lived in their local community for more than 180 days during the previous 12 months.

Trained interviewers conducted face-to-face interviews at the respondents’ homes and local community centers. All of the respondents signed an informed consent form before the start of the survey. They were also informed of their right to withdraw from the study at any time. A total of 853 people were interviewed, and 800 people completed the interview. The Short Portable Mental Status Questionnaire (SPMSQ) was used to assess the respondents’ cognitive function [18]. A power analysis using the G-Power computer program [19] indicated that a total sample of 89 people would be needed to detect moderate effects (f = 0.15) with 95% power using linear multiple regression. After removing observations with missing values for key variables, a total sample of 781 individuals was used in the analysis. A comparison between the full observations with imputed missing data (*N* = 799) and the reduced observations without missing data (*N* = 781) showed that they were not significantly different in any of the research variables (Please refer to the Additional file 1 for the detailed results).

This survey submitted a research proposal and related materials for research ethics review and was endorsed by the institutional review board of The University of Hong Kong (Reference No.: EA2003026).

### Measurements

#### Dependent variable

Depressive symptom was the study’s dependent variable. We used the 10-item Center for Epidemiologic Studies Depression Scale (CES-D-10), which has commonly been used to measure the levels of depressive symptoms among older respondents [20, 21]. This scale included negative-mood items, such as “I am troubled by some small things” and “I feel lonely,” items related to positive emotions, such as “I am happy” and “I am hopeful for the future,” and items related to somatic syndromes, such as “My sleep is not good.” Answers were assessed on a five-point Likert-type scale (1 = rarely or none of the time, 2 = not much of the time, 3 = almost half of the time, 4 = most of the time, 5 = almost every day). Two reverse questions were reverse coded. Summed scores represented the level of depressive symptoms (in a range of 0–50), with higher scores indicating higher levels of depressive symtoms. The Cronbach’s alpha estimate of CES-D-10 was 0.755 in this sample.

#### Independent variable

Oral health was the study’s independent variable and was measured by one single question: “What do you think of your current oral health condition (including your teeth or dentures, and your gums)?” The responses were assessed on a six-point scale (ranging from 1 = terrible to 6 = very good), so the higher the score was, the better the respondent’s oral condition was. Self-rating provides a simple and direct way of capturing perceptions of individual health and oral health that is valid, reliable, and cost-effective [22, 23].

#### Mediator

Daily dietary satisfaction was used as a mediator in this study, and it was measured by one single question: “Are you satisfied with your daily diet?” The responses were assessed on a five-point Likert scale (ranging from 1 = Very dissatisfied to 5 = Very satisfied). According to the existed literature, a single item is a direct, valid, reliable, and cost-effective measure [22], and research already exists on the use of single items to measure dietary satisfaction [24].

#### Moderator

Body mass index was treated as a moderator in this study, and the body mass index was calculated with SPSS software. Using each respondent’s self-reported height (in meters) and weight (in kilograms), body mass index was calculated by dividing the individual’s weight in kilograms by their height in meters squared. According to the World Health Organization criteria, body mass index values ranging from 18.5 to 25 are regarded as normal [25].

#### Covariates

We controlled for the respondents’ sociodemographic characteristics and health status in the model, selecting gender, age, marital status, education, income, number of chronic diseases, and self-rated health, because those characteristics are significantly associated with depressive symptoms [4–6]. Age, education, and income were defined as continuous measures. Self-rated health was assessed by using a five-point Likert scale. Sex was a dichotomous value (1 = male, 0 = female). Marital status was coded as “with partner” = 1 and “without partner” (i.e., single, widowed, or divorced) = 0. Finally, respondents were asked whether they had any of the 14 chronic diseases that are most common among older adults in China. The answers were recoded as binary variables (0 = no, 1 = yes). Summed scores were then used to represent the number of chronic diseases.

### Data analysis

The means and standard deviations (SD) of the continuous variables, together with the frequency and percentages of the categorical variables, were used to describe the variables. Path analysis conducted with Amos 24.0 software through the maximum likelihood method was performed to examine the model.

The model fit index included the root mean square error of approximation (RMSEA), the standardized root-mean-square residual (SRMR), the incremental-fit index (IFI), the adjusted goodness-of-fit index (AGFI), the goodness-of-fit index (GFI), the comparative fit index (CFI), and Chi-square divided by the degrees of freedom (CMIN/DF). As recommended in previous literature, values of RMSEA and SRMR smaller than 0.08, values of CMIN/DF smaller than 5, and values of IFI, AGFI, GFI, and CFI greater than 0.9 indicate an acceptable fit [26]. The bias-corrected bootstrap method was used to examine the significance of the mediation effect. Specifically, we used 10,000 bootstrap samples and determined the bias-corrected 95% confidence interval. A confidence interval that does not contain zero indicates that the effect being evaluated is significant [27]. The total effect, indirect effect, and direct effect were calculated through bootstrapping set at 10,000 samples. In addition, the percentage of the total effect that was explained by indirect pathways was calculated.

Finally, the moderated mediation model was tested by using the Hayes PROCESS macro version 3.5 software (Model 14) [28]. The 95% bias-corrected confidence interval from 10,000 resamples was generated by the bias-corrected bootstrapping method to examine the significance of the moderated mediation effect, and the Johnson-Neyman technique was used to visualize the moderating effect of body mass index in the relationship of dietary satisfaction and depressive symtoms. A significance level of 0.05 was used in the current study.

## Results

### Descriptive analysis

Descriptive characteristics of the respondents are presented in Table 1. More than half of the respondents were female (61%; *n* = 477), and more than half were married (78%; *n* = 609). Most of our older-adult respondents (96.5%; *n* = 754) reported that they were satisfied or very satisfied with their diet, with only 3.5% (*n* = 27) reporting that they were dissatisfied with their diet. Meanwhile, 48.3% (*n* = 377) of the study’s sample of older adults reported that their oral health was better than good, and 51.7% (*n* = 404) reported that their oral health was not good enough. In terms of educational attainment, 8.7% (*n* = 68) were illiterate, 16.3% (*n* = 127) had graduated from elementary school, 37% (*n* = 289) had graduated from middle school, 26.5% (*n* = 207) had graduated from high school, and 11.5% (*n* = 90) had graduated from college. The average age of the respondents was 71.33 years (SD = 7.03). Their mean score of depressive symptoms was 13.18 (SD = 4.41), indicating low levels of depressive symptoms. In addition, the respondents’ average body mass index was 24.68 (SD = 3.2), which according to the World Health Organization criteria was in the normal range. The respondents’ average monthly income was 5271.28 yuan (SD = 4047.2). Their average number of chronic diseases was 1.67 (SD = 1.65), and their mean score for their self-rated health was 3.56 (SD = 0.82).

**Table 1:** Descriptive analysis on the variables included in this study (*N* = 781)

	Mean	SD	N (Valid%)
**Gender**			
*Male*			304 (38.9%)
*Female*			477 (61.1%)
**Education**			
*Illiteracy*			68 (8.7%)
*Primary*			127 (16.3%)
*Junior high school graduation*			289 (37.0%)
*Graduated from high school*			207 (26.5%)
*University graduation*			90 (11.5%)
**Marital status**			
*Not being married*			172 (22%)
*Being married*			609 (78%)
**Dietary satisfaction**			
*Very dissatisfied*			1 (0.1%)
*Dissatisfied*			2 (0.3%)
*Fair*			24 (3.1%)
*Satisfied*			443 (56.7%)
*Very satisfied*			311 (39.8%)
**Oral health**			
*Very bad*			30 (3.8%)
*Poor*			144 (18.4%)
*Fair*			230 (29.4%)
*Good*			192 (24.6%)
*Pretty good*			108 (13.8%)
*Excellent*			77 (9.9%)
**Depressive symptoms**	13.18	4.41	
**Age**	71.33	7.03	
**Body mass index**	24.68	3.2	
**Income**	5271.28	4047.2	
**Number of diseases**	1.67	1.65	
**Self-rated health**	3.56	0.82	

*SD* Standard deviation

### Bivariate association analyses

Table 2 presents the results of the bivariate tests between the study variables, with depressive symptoms as the dependent variable. The results revealed that the socioeconomic variables were significantly related to depressive symptoms in our respondents. Table 2 shows that the elderly adults who were married had a lower level of depressive symptoms than those who were not married did. It was also found that relatively higher levels of education, higher incomes, higher scores for oral health, greater dietary satisfaction, higher self-rated health scores, and higher body mass index scores were related to lower levels of depressive symptoms. In addition, age and the number of diseases were found to be positively associated with depressive symptoms. However, gender was not found to make a difference in depressive symptoms in our sample.

**Table 2:** Bivariate test between depressive symptoms and other variables (*N* = 781)

Variables	Depressive symptoms		
	Mean (SD)	Test	*P*-Value
Gender		t = 0.34	0.737
*Male*	13.12 (4.23)		
*Female*	13.23 (4.52)		
Marital status		t = 2.21	0.027
*Not being married*	13.84 (5.31)		
*Being married*	13.00 (4.11)		
Age		*r* = 0.09	0.011
Education		*r* = −0.08	0.025
Income		*r* = − 0.10	0.004
Oral health		*r* = − 0.17	< 0.001
Dietary satisfaction		*r* = − 0.21	< 0.001
Body mass index		*r* = − 0.11	0.003
Number of diseases		*r* = 0.30	< 0.001
Self-rated health		*r* = −0.35	< 0.001

*SD* Standard deviation, *t* Independent t-test, *r* Pearson correlation tests

### The mediation analyses

Before the mediation analyses, multiple regression of oral health on depressive symptoms was conducted (hypothesis 1). The results showed a significant negative association between oral health and depressive symptoms (B = − 0.28, SE = 0.11, *p* < 0.05). Therefore, the first hypothesis was supported (the results were attached as Additional file 2), and the moderated mediation model test could then be continued. Table 3 presents the conditional indirect effect that oral health had on depressive symptoms through dietary satisfaction, with moderating by body mass index. The first part in Table 3 shows the effect of oral health on the mediator (dietary satisfaction). The results reveal that relatively better oral health was associated with a higher level of dietary satisfaction (path a; B = 0.06, SE = 0.03, 95%CI [0.01, 0.11]). The second part in Table 3 shows the effect of oral health on depressive symptoms, after taking into account all of the covariates, mediators, and moderated mediation effects. The results indicated that oral health had a significant direct effect on depressive symptoms (path c) even after including the covariates, mediators, and moderators (B = -0.22, SE = 0.11, 95%CI [− 0.44, − 0.01]). The results also revealed that relatively higher dietary satisfaction was related to lower levels of depressive symptoms (path b; B = − 0.61, SE = 0.15, 95%CI [− 0.89, − 0.32]). In addition, an interaction effect between dietary satisfaction and body mass index on depressive symptoms was found to be statistically significant (path d; B = 0.47, SE = 0.15, 95%CI [0.18, 0.76]). Moreover, the number of chronic diseases that a respondent had was found to be positively associated with depressive symptoms (B = 0.53, SE = 0.10, *p* = 0.03), whereas income (B = -0.34, SE = 0.16, *p* < 0.001) and self-rated health (B = -1.21, SE = 0.20, *p* < 0.001) were negatively associated with depressive symptoms, and no significant association was found between depressive symptoms and the other covariates. The *R*^2^ was 0.22 in this model, which means that the model explained 22.2% of the variance in depressive symptoms.

**Table 3:** Direct and indirect effect of oral health on depressive symptoms through mediator (Dietary satisfaction) conditionally in the level of moderator (BMI) (*N* = 781)

Path: Predictor	B	SE	t	*P*-Value	*R*^2^
	Dietary satisfaction as outcome				0.22
a: Oral health	0.06	0.03	2.19	0.029	
	Depressive symptoms as outcome				
c: Oral health	-0.22	0.11	−2.00	0.046	
b: Dietary satisfaction	−0.61	0.15	−4.20	< 0.001	
Body mass index	−0.68	0.15	−4.44	< 0.001	
d: Dietary satisfaction*BMI	0.47	0.15	3.21	< 0.001	
Covariates					
Education	−0.15	0.14	−1.11	0.27	
Marital status	−0.08	0.38	−0.21	0.83	
Gender	0.12	0.29	0.40	0.69	
Age	0.01	0.02	0.41	0.68	
Income	−0.34	0.16	−2.21	0.03	
Number of diseases	0.53	0.10	5.50	< 0.001	
Self-rated health	−1.21	0.20	−6.20	< 0.001	

*B* Unstandardized regression coefficient, *SE* Standard error

Oral health was found to have a significant total effect, direct effect, and indirect effect on depressive symptoms, and the results are presented in Table 4 (total effect: B = − 0.28, SE = 0.11, 95%CI [− 0.501, − 0.055], direct effect: B = − 0.24, SE = 0.11, 95%CI [− 0.460, − 0.017], and indirect effect: B = − 0.04, SE = 0.02, 95%CI [− 0.09, − 0.002]). Thus, dietary satisfaction partially mediated the relationship between oral health and depressive symptoms, and the indirect effect accounted for 14.28% of the total effect.

**Table 4:** The total, direct and indirect effect of oral health on depressive symptoms

Total and direct and indirect effect of Oral health on depressive symptoms			
Total effects of Oral health on depressive symptoms			
B	SE	95% Confidence Interval	*P*-Value
−0.28	0.11	[− 0.501, − 0.055]	< 0.05
Direct effects of Oral health on depressive symptoms			
B	SE	95% Confidence Interval	*P*-Value
−0.24	0.11	[−0.460, − 0.017]	< 0.05
Indirect effects of Oral health on depressive symptoms			
B	SE	95% Confidence Interval	*P*-Value
−0.04	0.02	[−0.09, − 0.002]	< 0.05

*B* Unstandardized regression coefficient, *SE* Standard error

### The moderated mediation analyses

**Fig. 1:**
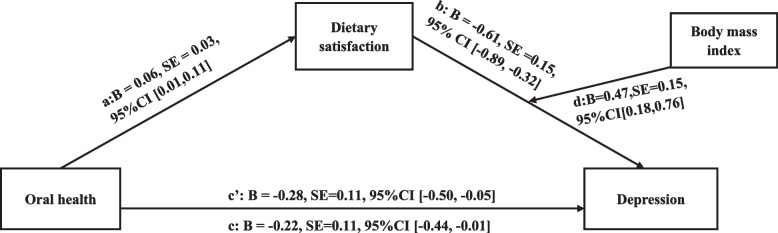
The moderated mediation model including oral health, dietary satisfaction, BMI and depressive symptoms. CMIN = 76.394, DF = 26, CMIN/DF = 2.938, GFI = 0.984, AGFI = 0.953, IFI = 0.939, CFI = 0.936, RMSEA = 0.05, SRMR = 0.037. *Notes:* c = direct effect, c’ = total effect; B=Unstandardized regression coefficient; SE = Standard error; BMI = Body Mass Index; CI = confidence interval; CMIN = Chi-Square; DF = Degree of freedom; GFI = Goodness of fit index; AGFI = Adjusted goodness of fit index; IFI = Incremental fit index; CFI = Comparative fit index; RMSEA = Root mean square error of approximation; SRMR = Standardized root mean square residual; The covariates were age, gender, education, marital status, income, number of disease and self-rated health

A structural equation model using AMOS software was established, and the details of the moderated mediation model are shown in Fig. 1. The moderating effect of body mass index between dietary satisfaction and depressive symptoms was significant (path d; B = 0.47, SE = 0.15, 95%CI [0.18, 0.76]). The fit indexes indicated an adequate model fit (CMIN/DF = 2.938, GFI = 0.984, AGFI = 0.953, IFI = 0.939, CFI = 0.936, RMSEA = 0.05, SRMR = 0.037).

**Fig. 2:**
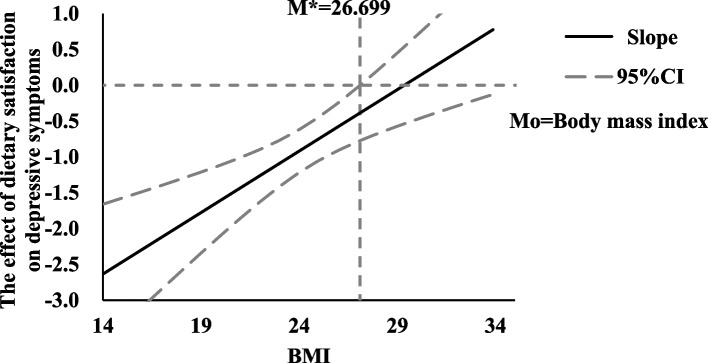
Johnson-Neyman diagram of the moderator effect of BMI on the association between dietary satisfaction and depressive symptoms. *Notes:* Mo = Moderator variable; M* = Critical value of moderator variable; BMI = Body Mass Index; 95% CI = 95% confidence interval

The result of the moderator effect, as visualized by the Johnson-Neyman technique, is shown in Fig. 2. As this figure shows, the effect of dietary satisfaction on depressive symptoms was moderated by body mass index, and the critical value of the moderating variable was 26.699, which means that when the body mass index was higher than 26.699, the effect of dietary satisfaction on depressive symptoms was not significant, because the confidence interval contained zero. When the value of body mass index was lower than the critical value, however, the confidence interval did not contain zero, thus indicating that the effect of dietary satisfaction on depressive symptoms was significant, and the slope of the effect was negative, meaning that the greater the individual’s dietary satisfaction was, the less depressed they would have been. Moreover, the slope of the line for the effect of dietary satisfaction on depressive symptoms decreased as body mass index increased, indicating that the effect of dietary satisfaction on depressive symptoms was much greater in people with relatively lower body mass index than it was in people with higher body mass index.

## Discussion

This study is the first to examine the relationship between oral health and depressive symptoms in the Chinese older population by using a moderated mediation model. In this model, we assumed that an association between oral health and depressive symptoms could occur directly and/or indirectly via daily dietary satisfaction as a mediator. We also hypothesized that body mass index moderated the path between daily dietary satisfaction and depressive symptoms.

As expected, this study’s results indicated a significant negative association between oral health and depressive symptoms, after including covariates, mediators, and moderators (B = − 0.22, SE = 0.11, 95%CI [− 0.44, − 0.01]). Specifically, the better the oral health of the older adults was, the lower their risk of depressive symptoms was. These findings echoed those of studies conducted in non-Chinese communities [7, 8, 29] that have explored relationships between oral health (e.g., tooth loss, edentulism, chewing ability, et al.) and depression. However, the current study added new empirical evidence to that research area by adopting a large sample in an investigation in Chinese communities.

Moreover, this study also confirmed the indirect effect of oral health on depressive symptoms through dietary satisfaction. The results indicated that dietary satisfaction partially mediated the relationship between oral health and depressive symptoms (B = − 0.04, SE = 0.02, 95%CI [− 0.09, − 0.002]) and that the indirect effect accounted for 14.28% of the total effect. Those results expand the accumulated knowledge, which had indicated oral health to be a risk factor for depressive symptoms by pointing out a mediation mechanism within it. Specifically, better oral health was positively related to better daily dietary satisfaction, and the latter was associated with a lower risk of being depressed. That finding not only agreed with available research suggesting that older people’s food selection and nutritional in-taking were largely affected by oral health status [7, 9–11], but it also revealed the vital roles of food and appetite in older people’s mental health in Chinese culture. There were two possible mechanisms explaining the mediation effect of daily dietary satisfaction on the relationship between oral health and depressive symptoms. Firstly, Chinese people are particularly passionate and serious about food and daily diet [30, 31]. In terms of recipes, the traditional aesthetic of Chinese food arises from taste sensitivity to diverse ingredients and in turn extends to the senses of smell and sight, and finally to mental consciousness [32]. Thus, older adults with poor oral health may have limited choices on taste, fragrance and character of cuisine of their daily diet, which brings less satisfaction from their daily food consumption, consequently decreasing mental pleasure. Secondly, it is worth mentioning the social functions of food in Chinese communities. Food plays an important part in family reunions, gatherings of friends, and festival celebrations, and is a vehicle for expression of friendship, for smoothing social intercourse, and for showing concern. Thus, it is not difficult to infer that older individuals with poor oral health may also be less socially integrated in family reunions and gatherings of friends, both of which would decrease satisfaction with their daily diet and increase their probability of suffering depressive symptoms.

A further moderated mediation effect was demonstrated in this research by revealing that the path between daily dietary satisfaction and depressive symptoms was moderated by body mass index. The results showed that when body mass index exceeded the threshold of 26.699 that was identified in the Johnson-Neyman results, the effect of daily dietary satisfaction on depressive symptoms was not significant (notably, our cutoff was very near the criterion from the World Health Organization that people with body mass index higher than 25 are regarded to be overweight [33]). When the value of body mass index in our study was smaller than 26.699, the effect of dietary satisfaction on depressive symptoms was negatively significant. Thus, in contrast to previous research indicating that overweight older adults were more likely than others in their age group to be depressed [12–16], our study has provided a new conclusion by emphasizing that older adults with a fit weight may actually be more vulnerable to depressive symptoms, at least if they are not satisfied with their daily diet. The different reactions in terms of older individuals with lower body mass index and those with higher body mass index supported previous research documenting dietary intake patterns vary with body mass index [17, 34, 35]. Fitter older adults usually prefer a dietary intake pattern characterized by higher consumption levels of cereals, nuts, vegetables and fruits consumption (which required healthier oral health conditions) than those in the obese group [14]. The findings of moderated mediation effect may suggest that older individuals with lower body mass index would derive less satisfaction from their daily diet when their oral health becomes worse and would experience more changes in food preference and even life style, which causes negative emotions and cognitions, in turn having an adverse effect on their mental health.

The present research contributes to the accumulated knowledge by providing a new and comprehensive perspective that broadens our understanding of the unique experience that connects oral health, daily dietary satisfaction, and depressive symptoms among Chinese older adults. The findings present evidence for policymakers and researchers to use in identifying risk factors that influence older adults’ aging process, planning related social services in further studies, and designing services. We proposed the policy and intervention implications, as follows:

Firstly, as indicated in this research, oral health is an important contributor to depressive symptoms among older people, and therefore we suggest that designers and social service officers of health management programs institute regular oral health monitoring and protection plans. In addition, oral health parameters should be included in future studies and projects evaluating older individuals’ well-being. Secondly, because we found that individuals’ daily dietary satisfaction can have a strong influence on the mental health status of Chinese older adults, and that one’s body mass index can moderate that effect, efforts to improve daily dietary satisfaction, such as through enhancing awareness of people’s dietary habits and preferences, improving their dietary diversity, and guiding their nutritional intake, should be regarded as effective strategies for reducing older adults’ poor-oral-health-related risks for depressive symptoms, especially for the fitter older individuals in this group. This study was subject to some limitations. First, we did not explore any causal relationship, because this research adopted a cross-sectional design. In future studies, a longitudinal approach will be needed to address that issue. Second, due to limited research resources, we had to employ a quota sampling approach, which may have limited the generalizability of the findings. In addition, although the data collection process was anonymous, the main variables in this study were self-reported, which probably lead to response bias. Further studies are encouraged to use alternative data collection and analysis methods in order to improve statistical accuracy. Last, CESD-10 was employed to measure depressive symptoms in this study, which only acted as a screening tool. We strongly recommend further studies verify our present findings among older adults with clinical diagnosis of depression.

## Conclusions

This study examined the relationship between oral health and depressive symptoms in Chinese older adults, using a moderated mediation model. A significant negative association between oral health and depressive symptoms has been indicated, and dietary satisfaction partially mediated the relationship between oral health and depressive symptoms. The path was moderated by body mass index, and the effect of dietary satisfaction on depressive symptoms was much greater in people with relatively low body mass index.

The present study is among the first study to show evidence for policymakers and researchers that strategies to enhance oral health and daily dietary satisfaction could be important for preventing depressive symptoms in Chinese older adults, and especially for the relatively fitter older groups with lower body mass index.

## Supplementary Information

**Additional file 1.**

**Additional file 2.**

## Data Availability

The datasets used and/or analysed during the current study are available from the corresponding author on reasonable request.

## Abbreviations

CHARLS: China Health and Retirement Longitudinal Study
BMI: Body Mass Index
SPMSQ: Short Portable Mental Status Questionnaire
CES-D-10: The 10-item Center for Epidemiologic Studies Depression Scale
JN: Johnson-Neyman
SD: Standard deviation
RMSEA: The root mean square error of approximation
SRMR: The standardized root-mean-square residual
IFI: The incremental-fit index
AGFI: The adjusted goodness-of-fit index
GFI: The goodness-of-fit index
CFI: The comparative fit index
CMIN/DF: Chi-square divided by the degrees of freedom
