# Supplementary material for: Oral health and depressive symptoms among older adults in urban China: a moderated mediation model analysis
Source: BMC Geriatr. 2022 Oct 28;22:829. doi: 10.1186/s12877-022-03542-1 (PMC9617299; doi:10.1186/s12877-022-03542-1)
Supplement: Supplementary file 1 — Additional file 1. [file 12877_2022_3542_MOESM1_ESM.docx]

**Additional file 1:**

Comparison of the full observations and the reduced observations with missing data

| Variables | Full sample(N=799) | | | Reduced sample(N=781) | | | P Value |
| --- | --- | --- | --- | --- | --- | --- | --- |
|  | Percentage | | Mean | Percentage | | Mean |  |
| Male | 38.80% |  | | 38.90% |  | | >0.1 |
| Being married | 77.90% |  | | 78.00% |  | | >0.1 |
| Illiteracy | 8.90% |  | | 8.70% |  | | >0.1 |
| Primary | 16.04% |  | | 16.30% |  | | >0.1 |
| Junior high school graduation | 36.47% |  | | 37.00% |  | | >0.1 |
| Graduated from high school | 26.94% |  | | 26.50% |  | | >0.1 |
| University graduation | 11.65% |  | | 11.50% |  | | >0.1 |
| Income |  | 5270.65 | |  | 5271.28 | | >0.1 |
| Oral health |  | 3.56 | |  | 3.56 | | >0.1 |
| Dietary satisfaction |  | 4.36 | |  | 4.36 | | >0.1 |
| Body mass index |  | 24.68 | |  | 24.76 | | >0.1 |
| Number of diseases |  | 1.62 | |  | 1.61 | | >0.1 |
| Self-rated health |  | 3.56 | |  | 3.56 | | >0.1 |
| Age |  | 71.39 | |  | 71.33 | | >0.1 |
