# Supplementary material for: Oral health and depressive symptoms among older adults in urban China: a moderated mediation model analysis
Source: BMC Geriatr. 2022 Oct 28;22:829. doi: 10.1186/s12877-022-03542-1 (PMC9617299; doi:10.1186/s12877-022-03542-1)
Supplement: Supplementary file 2 — Additional file 2. [file 12877_2022_3542_MOESM2_ESM.docx]

**Additional file 2:**

Multiple regression results of oral health on depressive symptoms

|  | Model 1 | | | | Model 2 | | | |
| --- | --- | --- | --- | --- | --- | --- | --- | --- |
|  | B | SE | t | P-Value | B | SE | t | P-Value |
| Constant | 17.258 | 2.023 | 8.531 | <0.001 | 18.003 | 2.039 | 8.827 | <0.001 |
| Education | -0.094 | 0.144 | -0.653 | 0.514 | -0.079 | 0.144 | -0.551 | 0.582 |
| Marital status | -0.118 | 0.393 | -0.300 | 0.764 | -0.092 | 0.391 | -0.235 | 0.814 |
| Gender | 0.227 | 0.308 | 0.737 | 0.461 | 0.181 | 0.307 | 0.588 | 0.557 |
| Age | 0.007 | 0.022 | 0.335 | 0.738 | 0.007 | 0.022 | 0.296 | 0.767 |
| Income | -0.391 | 0.161 | -2.429 | <0.05 | -0.388 | 0.161 | -2.415 | <0.05 |
| Self-rated health | -1.438 | 0.198 | -7.266 | <0.001 | -1.358 | 0.200 | -6.795 | <0.001 |
| Number of diseases | 0.493 | 0.098 | 5.009 | <0.001 | 0.478 | 0.098 | 4.863 | <0.001 |
| Oral health |  |  |  |  | -0.278 | 0.114 | -2.446 | <0.05 |
| R-Square | 0.162 | | | | 0.169 | | | |

*Notes:* B=Unstandardized regression coefficient; SE = Standard error.
